# Node Localization Method in Wireless Sensor Networks Using Combined Crow Search and the Weighted Centroid Method

**DOI:** 10.3390/s24154791

**Published:** 2024-07-24

**Authors:** Suresh Sankaranarayanan, Rajaram Vijayakumar, Srividhya Swaminathan, Badar Almarri, Pascal Lorenz, Joel J. P. C. Rodrigues

**Affiliations:** 1 Department of Computer Science, King Faisal University, Al Hofuf P.O. Box 400, Saudi Arabia; baalmarri@kfu.edu.sa; 2 Department of Networking and Communications, School of Computing, SRM Institute of Science and Technology, Chennai 603203, India; 3 Department of Data Science and Business Systems, School of Computing, Faculty of Engineering and Technology, SRM Institute of Science and Technology, Kattankulathur, Chennai 603203, India; srividhs1@srmist.edu.in; 4 Institute de Recherche en Informatique, Mathématique, Mathématiques, Automatique et Signal, University of Haute Alsace, 68100 Colmar, France; pascal.lorenz@uha.fr; 5 Higher School of Technology, Amazonas State University, Manaus 69050-020, AM, Brazil; joeljr@ieee.org

**Keywords:** localization, anchor nodes, crow search algorithm, range free

## Abstract

Node localization is critical for accessing diverse nodes that provide services in remote places. Single-anchor localization techniques suffer co-linearity, performing poorly. The reliable multiple anchor node selection method is computationally intensive and requires a lot of processing power and time to identify suitable anchor nodes. Node localization in wireless sensor networks (WSNs) is challenging due to the number and placement of anchors, as well as their communication capabilities. These senor nodes possess limited energy resources, which is a big concern in localization. In addition to convention optimization in WSNs, researchers have employed nature-inspired algorithms to localize unknown nodes in WSN. However, these methods take longer, require lots of processing power, and have higher localization error, with a greater number of beacon nodes and sensitivity to parameter selection affecting localization. This research employed a nature-inspired crow search algorithm (an improvement over other nature-inspired algorithms) for selecting the suitable number of anchor nodes from the population, reducing errors in localizing unknown nodes. Additionally, the weighted centroid method was proposed for identifying the exact location of an unknown node. This made the crow search weighted centroid localization (CS-WCL) algorithm a more trustworthy and efficient method for node localization in WSNs, with reduced average localization error (ALE) and energy consumption. CS-WCL outperformed WCL and distance vector (DV)-Hop, with a reduced ALE of 15% (from 32%) and varying communication radii from 20 m to 45 m. Also, the ALE against scalability was validated for CS-WCL against WCL and DV-Hop for a varying number of beacon nodes (from 3 to 2), reducing ALE to 2.59% (from 28.75%). Lastly, CS-WCL resulted in reduced energy consumption (from 120 mJ to 45 mJ) for varying network nodes from 30 to 300 against WCL and DV-Hop. Thus, CS-WCL outperformed other nature-inspired algorithms in node localization. These have been validated using MATLAB 2022b.

## 1. Introduction

Localization methods are used in many real-world applications, including in the industrial realm [1] (for inventory stock identification), in underwater environments, and for outdoor activities [2]. It is used in classic global positioning systems (GPS) to achieve a high level of localization accuracy in outdoor environments. It is impracticable to equip every sensor node in a large-scale wireless sensor network (WSN) with a GPS device due to the costs and power requirements. In a WSN, node localization requires data analysis and node position information. Localization techniques can help to resolve this issue. One technique for identifying a sensor node in WSNs is beacon-based localization [3]. The technique entails choosing one or more beacon nodes whose positions are known and calculating the received signal strength (RSS) between these beacons and the target node. The target node can then determine its location based on the measured distances or RSS values, using trilateration or multilateration [4].

When selecting beacon nodes, several factors should be considered, including the number of beacons needed, their placement, and their communication capabilities. In general, additional beacon nodes will improve accuracy but also make the localization system more complex and expensive [5]. Several techniques can be used for node localization in WSN, including anchor-based localization [6,7], range-free localization [8], hybrid localization [9], and multidimensional scaling.

When implementing node localization in a WSN, several factors should be considered, including the number of anchors needed, their placement, and the communication capabilities of the nodes [10]. The accuracy and efficiency of the localization algorithm also play a crucial role in determining the overall performance of the localization system. Additionally, the energy consumption of the localization process should be considered, as sensor nodes typically have limited energy resources [11]. Selecting and implementing a technique should be based on the required application and resources available in the network. One method for locating a target node is triangulation, which uses the distance from several anchor nodes [12]. The drawback of this technique is that it requires a high degree of accuracy in distance measurement and can be affected by multipath fading. Multidimensional scaling (MDS) has been proposed [13], which maps the distance between nodes in a low-dimensional space. Although it is computationally difficult and involves several distance measurements, it makes it easier to locate a target node.

With a map of the network environment and a target node′s specific characteristics, fingerprinting includes locating the target node. It requires a lot of prior knowledge about the network environment and can be affected by changes in the environment [14]. A nature-inspired optimization technique called particle swarm optimization (PSO) uses a swarm of particles to search for the best solution, but it is computationally intensive and requires a lot of processing power [15]. The requirements and constraints of the network environment determine the localization approach to be used. Each of these tactics has its advantages and disadvantages. The evolutionary [16] and salp swarm algorithms [17] are two examples of nature-inspired algorithms that are used to solve the node localization issues in isotropic WSNs. Anisotropic WSNs suffer localization problems, even though they are employed in practical applications. The multilateration approach to position calculation and hop-based range-free [18] distance measuring are the foundation for the localization algorithms created to overcome anisotropic WSN localization challenges. Most nature-inspired algorithms [19] offer more benefits than conventional optimization techniques, including the capacity to efficiently explore a vast solution space and locate optimal solutions in the presence of noise. However, they can also be computationally and time demanding, requiring a lot of processing power to solve. Furthermore, these methods might be sensitive to parameter selection, which might affect the precision and dependability of the localization findings.

Therefore, in this paper, we have proposed the crow search-based weighted centroid localization (CS-WCL), which is an improvement over other nature-inspired algorithms used for localization. CS-WCL is a two-step process. In the first step, the crow search optimization is analyzed, and the best three anchor nodes are selected. In the second step, the selected anchor nodes participate in the localization process using the weighted centroid method to estimate the location of the unknown nodes. This algorithm aims to provide a more accurate localization solution for unknown nodes in WSNs by considering the trade-off between searchability and the change of awareness probability. The proposed strategy outperformed previously implemented soft computing localization algorithms in most of the simulated network topologies. The paper contributes by:

- Optimizing the anchor node selection using the crow search algorithm.
- Identifying the location of unknown nodes’ using weighted centroid localization.
- Validating and comparing CS-WCL to WCL and distance vector (DV)-Hop for varying numbers of nodes.

### 1.1. Motivation

Node localization in a WSN is very challenging due to the number and placement of anchors and the communication capabilities of the nodes. Moreover, the energy requirement of the localization process is very important, as sensor nodes typically have limited energy resources. Also, the biological inspired algorithms previously employed for localization are associated with higher localization errors and a greater number of beacon nodes for small- to large-scale networks. An improved localization algorithm called the CS-WCL, which reduces localization error for varying communication radii and beacon nodes, as well as energy consumption, has addressed these.

### 1.2. Significance of the Proposed Work

Anchor nodes are helpful for node localization in WSNs. Instead of taking all the anchors in the region, we employed the crow-based search optimization method to select the best anchor nodes. The best anchor nodes, selected by optimization, participated in the localization process using the weighted centroid method for estimating the location of the unknown nodes. The major advantage of this algorithm is that it provides a more accurate localization solution for unknown nodes in WSNs by considering the trade-off between the searchability and change of awareness probability. The major significance of the proposed algorithm is its accurate localization of unknown nodes with a minimum number of anchor nodes chosen, resulting in reduced localization error and energy consumption in comparison to previously implemented nature-inspired localization algorithms.

The remaining part of the paper is divided into the following sections. The related work on WSN localization techniques is presented in Section 2. Section 3 discusses the proposed work, which is the CS-WCL method for selecting the best anchor nodes, using the weighted centroid method for localization with the conceptual block diagram, the crow search optimization process, and WCL, with respective algorithms and flow charts. Section 4 discusses the implementation methodology, with simulation setup and performance metrics followed by the implementation of the proposed method that is CS-WCL, against DV-Hop and WCL, in terms of localization error, total data packets, and energy consumption, along with a comparative analysis of previous literature. The same has been tabulated and is shown through graphs. Section 5 concludes the paper, with recommendations for future work.

## 2. Literature Review

The algorithm for localization findings in various networks is fairly accurate and reasonable. However, in random sensor networks with uneven density, there is significant localization inaccuracy. As the amount of cumulative error rises, the minimum hop-count from the localization node to the beacon node decreases. Since the error is directly proportional to the hop-count value, the node localization coordinates are calculated using the estimated distance, with a significant error. This section will give a detailed review of various related work about node localization in WSN.

The WSN node localization approach called MA*-3DDV-Hop enhances the DV-Hop algorithm’s poor three-dimensional (3D) accuracy by adjusting the average distance per hop error and optimizing the hop-count values [20]. Additionally, the approach overcomes issues with premature and poor convergence by using the non-dominated sorting genetic algorithm (NSGA-II) to locally optimize coordinates and produce a Pareto optimal solution. Due to their quick convergence, effective memory usage, and capacity for producing good outcomes in real-world problems, optimization algorithms are well liked in WSN.

Sharma and Kumar, in their research work, proposed a method for finding an optimal hop size using the line search algorithm and optimizing the distance error using the genetic algorithm. This ultimately increased the accuracy of DV-Hop localization. It was done by employing only the selected anchor nodes [21]. The concept of co-planarity is useful for locating the anchor nodes that are used to localize unknown nodes because the model that was provided is an algorithm for 3D localization. These anchor nodes have been chosen to take part in the process of localizing the unknown nodes. The localization accuracy is unquestionably improved because of the suggested improvements; however, this comes at the expense of an increased computing burden for the optimization algorithms.

Messous et al. [22] presented their enhanced recursive DV-Hop technique. With the aid of a pair of polynomial coefficients, a connection was created between the number of hops taken and distance traveled. The inclusion of an error correction component resulted in an improvement in this connection. Additionally, localization was carried out using the least-squares method, which makes use of the enhanced distance estimate. The least-squares method is carried out recursively by selecting a small number of anchor nodes at random throughout each iteration. The challenge with this work is that the proposed technique behaves unpredictably if a random selection of anchor nodes is made for each iteration, and the iterative execution of the least square method would incur more complexity in computation.

Adaptive WCL (A-WCL) [23], which employs a link quality indicator (LQI), is proposed in this work. The algorithm uses the LQI difference as the weight rather than to increase precision. A-WCL uses quadratic weights to attain the same peer accuracy as WCL but does not require the intricate WCL calculation. Although it has a good strategy for increasing WCL when communication ranges change, the cutoff for the minimum LQI changes drastically, which is one of its challenges. Herein, an approach that enhances WCL in an intelligent ubiquitous environment by employing dynamic weighting parameters (which consider the RSSI of anchors where the method offers an improvement) is presented. The algorithm only requires WCL from the surrounding environment and can attain greater localization accuracy. The algorithm uses various adaptation degrees in various sub-regions. After segmenting the region composed of anchors into numerous sub-regions, the accuracy of WCL increases [24].

The authors provided a strategy that computes a weighting factor to enhance WCL and employs a global localization error as a corrective mechanism to adjust the initial localization result. To increase localization accuracy with global localization error factors, the method raises the bar for even distribution and high anchor densities. However, it has a limitation, which is that it is impossible to precisely obtain the global factors in a scenario where the region has various sub-regions with various location error factors [25].

The authors of this work used weights that have been modified by the degree of adaptation. To increase localization accuracy, they put forth the modified WCL algorithm, which modifies the weight structure but still relies on adjusting the degree to get the best localization accuracy. We are aware that the localization to the anchor has the greatest influence when it is the closest and the adaptation degree is higher. Therefore, the exact influence cannot be determined by adjusting the weights [26].

Several studies have been conducted involving placing a movable anchor with GPS capabilities into a sensing area and occasionally broadcasting the anchor′s most recent geometric coordinates [27]. The movable anchor node′s coordinates are gathered by the other sensor nodes. Later, the sensor nodes select three of the movable anchor node′s non-collinear coordinate points and use various mechanisms to estimate position. Numerous localization techniques have been developed based on this idea. In one study [28], the author created a range-free localization method based on a geometric theory on the perpendicular bisector of a chord in a virtual circle. The technique used a mobile anchor that moves through a sensing region while periodically broadcasting its coordinates. Nearby sensor nodes recorded the coordinates of the anchor to draw a chord on their communication range. This process was repeated until the sensor node had three moving anchor coordinate points or more within its communication range, resulting in the construction of two chords. The perpendicular bisector of these two chords was then used to determine where the sensor nodes were located.

The maximum-likelihood, min-max, and trilateration algorithms [29] were also developed, which resulted in significant inaccuracy. They fully utilize the position and distance of nearby anchor nodes and then use k-means to eliminate the results with large errors. K-means clustering uses a randomly chosen starting cluster center and may go several rounds before reaching a stable state. Here, there is a possibility that the ultimate state is not ideal. The localization problem is NP-hard in general, and it is crucial to quickly locate sensor nodes, especially for highly sensitive applications like military operations. The computing time required to find sensor nodes has decreased due to a stochastic process.

The trust-based beacon node localization algorithm [30] is a technique used for localization in underwater WSNs that uses a combination of trust-based and nature-inspired meta-heuristic strategies to improve the accuracy and efficiency of the localization process. It uses trust-based techniques to identify and select trustworthy beacon nodes for localization. Trust is computed based on the node′s past behavior and communication patterns. This helps to eliminate the effect of false or malicious nodes in the localization process. The nature-inspired meta-heuristic strategies used in this algorithm include techniques such as PSO and ant colony optimization. The use of these nature-inspired meta-heuristic strategies improved the accuracy and efficiency of the localization process by effectively exploring the search space and finding the optimal solution. Additionally, using trust-based techniques, the algorithm can also improve the security and reliability of the localization process.

The authors of this work have proposed an error minimization protocol for the localization of sensor nodes in WSNs to enhance the accuracy and efficiency of the localization process [31]. Both RSS and time of arrival (TOA) are used to accurately estimate the range of nodes in a 3D application area. The protocol leverages the anchor node′s location, which is presumably already known, to restrict the received signal to line-of-sight (LOS) and single or double reflection. Consequently, localization errors for non-LOS (NLOS) signals are reduced. The protocol employs the geometrical relationship between the anchor and sensor nodes for both LOS and NLOS signals to first address the misclassification problem. To accomplish precise range estimation, it starts at the wrong node position and gradually shrinks the 3D space with each iteration.

The authors of this work have proposed a modified Archimedes optimization (MAOADV) Distance Vector Hop algorithm to improve the localization accuracy of the DV-Hop algorithm in WSNs [32]. By combining chaotic mapping and PSO into the Archimedes optimization method, the technique increases the initial population diversity. The algorithm′s capability for global convergence and speed is enhanced. To increase the localization accuracy, it then swaps out the least-squares component of the DV-Hop localization technique for the MAOA. The DV-Hop technique for localizing nodes in WSNs has been improved by the genetic technique DV (GADV) [33] Hop algorithm. It uses a genetic algorithm to enhance node-positioning precision and reduce localization mistakes in WSNs. By limiting the viable region of the starting population and boosting its quality, the GADV-Hop algorithm outperforms the DV-Hop. Consequently, the GADV-Hop method may converge more quickly and detect unknown nodes more precisely. A comparative analysis has been shown in Table 1.

The major advantage and improvement of the proposed algorithm, CS-WCL, is its accurate localization of unknown nodes with a minimum number of anchor nodes chosen, resulting in reduced localization error and energy consumption in comparison to previously implemented nature-inspired localization algorithms.

## 3. Crow Search Weighted Centroid Localization Algorithm (CSWCL)—Proposed Work

**Figure 1:**
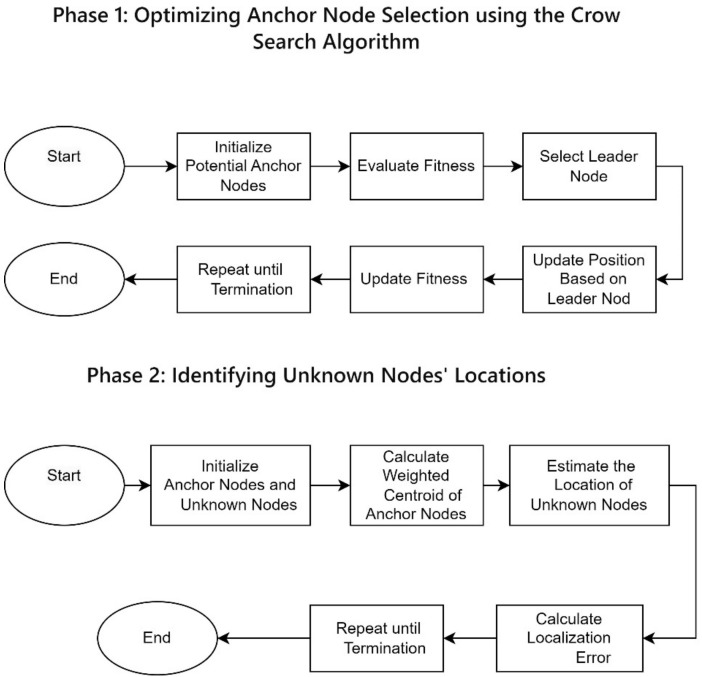
Crow search weighted centroid localization workflow.

**Figure 2:**
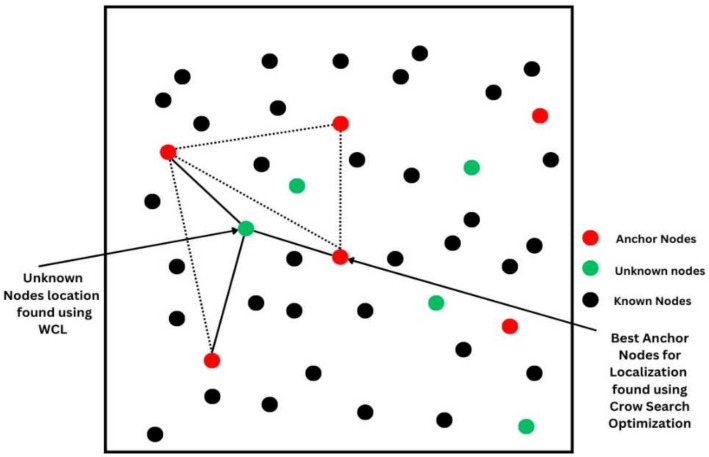
Conceptual diagram of CS-WLC.

The proposed CS-WCL involves two phases as shown in Figure 1, which shows the workflow architecture. The two phases are outlined below:

Figure 2 shows the CS-WCL method’s conceptual diagram. The details of the two phases of this method shown in the workflow architecture are explained below in Section 3.1 and Section 3.2.

### 3.1. Crow Search Optimization Process

The crow search optimization process begins with a randomly placed set of potential anchor nodes throughout the localization area. The crows of the group are *n*-dimension, and the algorithm states random vectors. All crows have their memory, where m_i_ = (m_i1_, m_i2_, …, m_in_). Crows do not know the source of food initially. In this algorithm, random vectors are chosen as anchor nodes [34] The *i*th crow is allocated with a random vector, i = 1, 2, …, n, which is attained from Equation (1).

(1) $$x_{i}=(\left(x_{i},1\right),\left(x_{i},2\right))\ldots (\left(x_{i},n\right))$$

The potential anchor node with the highest fitness value is chosen as the “leader” node. The other potential anchor nodes imitate the foraging behavior of the crows by moving toward the leader node. A search operator, based on the locations of the current potential anchor node and the leader node, determines their distance and direction of movement. A local search is performed by randomly perturbing the location of each potential anchor node within a specified range.

The fitness value is based on two important factors. Equation (2) tells us how far the anchor node is from other anchor nodes and helps us find its neighbors and the greatest amount of residual energy. Equation (3) is used to find the fitness function. The number of mobile nodes that a possible anchor node has access to is used to judge its fitness. The fitness value is found by adding the inverse distance between the possible anchor node and the visible mobile nodes.

Two main parameters determine the fitness value. The distance covered by the anchor node compared to other anchor nodes is specified in Equation (2) to identify the neighbors and maximum residual energy. The fitness function is attained using Equation (3). Each potential anchor node evaluates its fitness based on the number of mobile nodes it can communicate with. The inverse distance between the possible anchor node and visible mobile nodes is added to determine the fitness value.

(2) $$A_{XY}=N_{xy}+K_{xy}(N_{xy}-N_{zy})$$

$A_{XY}$ = *determines neighbor node* for $N_{xy}$, where $z=\left\{1,2,3\ldots \right\}$ and z≠x, *K* = *random number in the range* $\left[-1,1\right]$, y=1,2,3…V, z, and y values are arbitrarily selected.

$$P_{i}=\frac{{fit}_{x}}{\sum_{n=1}^{s}{fit}_{n}}$$

(3) $${fit}_{x}=\left\{\begin{array}{c} 1\\ 1+wi \end{array}\right.\;\;if\;zi\;\leq \;0\;1+wi\;,\;\;if\;zi>0$$

where $w_{i}$ is the fitness value given by

$$W_{i}=\frac{K_{1}\cdot N+K_{2}\cdot E_{res}}{K_{1}+K_{2}}$$

$K_{1}\;and\;K_{2}=weight\;constants$, N=node degree, $E_{res}=residual\;energy\;metrics$.

The potential anchor nodes compete based on their fitness values, and if a potential anchor node has a higher fitness value than the current leader node, it replaces the leader node.

Crow i randomly picks one Crow j from the group and follows it to where it has hidden its food. Crow i changes its position once it determines the location of Crow j. Equation (4) can be used to create a new position for Crow i. Equation (5) can be used to update the memory of crows.

(4) $$X_{i,k+1}=\left\{x_{i,k}+r_{i}\times {fl}_{i,k}\left(m_{j,k}-x_{i,k}\right)\;a_{j}\right\}\geq {AP}_{j,t}$$

$r_{i}\;and\;a_{j}\;denote\;random\;numbers\;with\;uniform$, distribution (between 0 and 1), ${AP}_{j,t}=Ppobability\;awareness\;of\;Crow\;j\;for\;iteration\;k$, ${fl}_{i,k}=Flight\;length\;for\;Crow\;i\;for\;the\;k_{th}\;iteration$, $M_{j,k}=Memory\;of\;Crow\;j\;for\;the\;k_{th}\;iteration$.

(5) $$m_{i,k+1}=\left\{\begin{array}{c} x_{i,k+1}\;\;\;\;\;\;\;\;\;\;\;\;\;f\left(x_{i,k+1}\right)≻f\left(m_{i,k}\right)\;\\ m_{i,k}\;\;\;\;\;\;\;\;\;\;\;\;\;\;\;\;\;\;\;\;\;\;\;Otherwise \end{array}\right\}$$

$m_{i,k}=crow^{\prime }s\;position\;j\;after\;k_{th}\;iteration$, $x_{i,k}=initial\;position$.

When a predefined stopping criterion is satisfied (for example, when a predetermined number of iterations have been finished), the algorithm ends. Using the crow search algorithm (CSA) to select the best anchor nodes, localization accuracy and reliability can be improved. The algorithm can also adapt to changes in the environment by dynamically selecting new anchor nodes based on the current mobile node locations. The maximum number of iterations is accomplished to estimate the best position of the crows. This way, the optimal solution is attained, i.e., the best anchor nodes are chosen. The algorithm for CSA is given below (Algorithm 1).
**Algorithm 1**: Crow Search Algorithm*1. **Initialize** the positions of N crows (anchor nodes) randomly in the group.**2. Assess the crows′ positions.**3. **Initialize** each crow′s (anchor nodes) memory.**4. **While** K < K_max:**   a. **For****   i = 1 to N (for all N crows in the group):**   i. Choose a crow (anchor nodes) at random to follow (for instance, Crow j).**   ii. **Define** an awareness* probability.*   iii. **If** a_j > AP_(j, t), then:**       x_(i, k + 1) = x_(i, k) + r_i × fl_(i, k) × (m_(j, k) − x_(i, k))**    **Else:****   x_(j, k + 1) = Taking random position in space.**   b. **Verify** the feasibility of new positions.**   c. **Evaluate** the new position of the crows.**   d. **Update** the memory of the crows.**   5. **End while.***

### 3.2. Weighted Centroid Localization

The recently created optimization algorithm called the CSA was motivated by the clever foraging methods used by crows. Range-free localization does not employ ranging to find and estimate the distance of unidentified nearby nodes and has been used in various applications. Using the minimum hop count and average distance, each sensor node determines how far it is from the beacon node. The distance to the beacon node is then calculated by multiplying the minimum hops by the average hop distance. It has three phases as outlined below. Using the coordinates of the known m anchor nodes chosen from the crow search optimization, node localization in WSNs is used to estimate the coordinates of N unknown nodes.

#### 3.2.1. Calculating a Minimum Number of Hops from Each Node to Each Anchor

To determine the smallest hop count, each beacon node sends out a beacon message that includes its position values and the hop count, which is initially set to zero. Following receipt, close-by nodes will increase this value and broadcast again. As a result, if the beacon message is received by the beacon node, it will log the sender′s coordinates and raise the hop count. In fact, the receiver node will check the hop number and add one if it receives a message from the same beacon node. When the new hop number is lower than the one it has stored, it will compare the two, modify its value, and send the message again with the new hop number. Otherwise, it will drop the message and not rebroadcast to its neighbors. After this step, all beacon and normal nodes will have the least hop count to every beacon node in the network.

In the first stage, all the anchor nodes send their information to the nodes connected in the network within l hop. The parameter ‘l’ is determined by the Equation (6).

(6) $$h_{mi}\leq l\leq h_{ma}$$

$h_{mi}$ = minimum hop value, $h_{ma}$ = maximum hop value.

Based upon the value of l, the power consumption is decided.

#### 3.2.2. Average Distance Per Hop

Every anchor node calculates the average distance per hop using Equation (7).

(7) $$Average\;distance\;per\;hop=\frac{\sum_{i=1i\neq j}^{m}\sqrt{{\left(x_{j}-x_{i}\right)}^{2}+{\left(y_{j}-y_{i}\right)}^{2}}}{\sum_{i=1i\neq j}^{m}h_{ji}}$$

*m* = total number of beacon nodes, *hij* = minimum hop count between beacon node i and j, (xi, xj) and (yi, yj) are coordinates of i and j.

Group cast: All the nodes connected to the anchor with the ‘l’ hops are joined together in a group. The anchor node group casts the message in the format of AMSG (*A_id_*,*X_loc_*,*Y_loc_*, *Hop_ui_*).

Instead of broadcasting the location information to the entire network, it groups casts to some set within a maximum of ‘l’ hop neighbors. It will reduce the control packet load. In the proposed algorithm, localization error is minimized because not all anchors are considered for localization. Each anchor node group casts its information to the group already formed.

##### 3.2.3. Location Information

The modified weighted metric is calculated by Equation (8). The computed location for the unknown node (Xun, Yun) using the CSWCL is given as per Equations (9) and (10).

(8) $$W_{j}={\left(\frac{\sum_{j=1}^{k}h_{uj}}{Kh_{uj}}\right)}^{\frac{r}{{AHS}_{i}}}$$

AHS_i_ = average hop size of nearest ‘i’ anchor nodes in the group.

(9) $$X_{un}=\frac{\sum_{j=1}^{k}W_{j}X_{j}}{\sum_{i=1}^{k}W_{j}}$$

(10) $$Y_{un}=\frac{\sum_{j=1}^{k}W_{j}Y}{\sum_{i=1}^{k}W_{j}}$$

**Figure 3:**
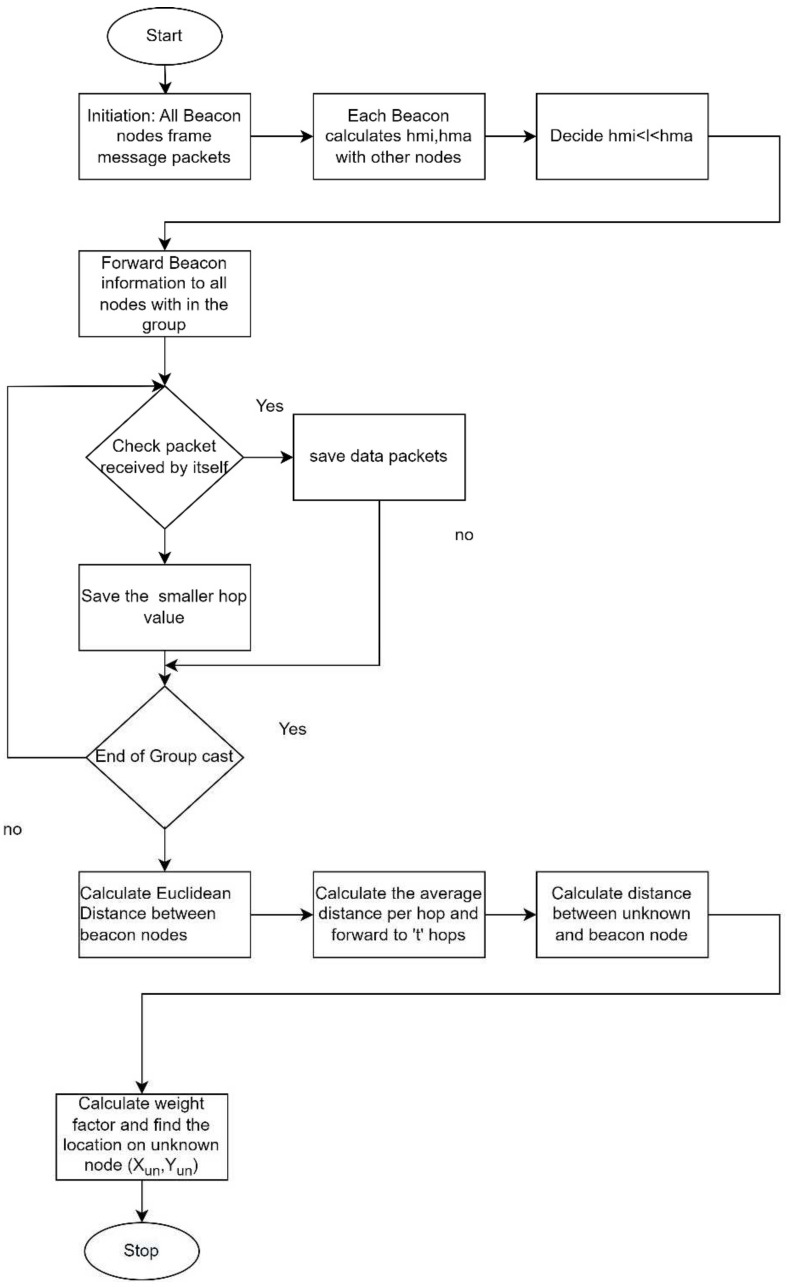
Localization process.

The complete flow chart of weighted centroid localization is outlined in Figure 3.

## 4. Implementation Results

The proposed CS-WCL was compared to other localization methods, including the DV-Hop and WCL methods. MATLAB was used for the simulation. The rationale behind comparing CS-WCL with WCL and DV-Hop lies in the need for a thorough benchmarking analysis. WCL and DV-Hop are widely recognized methods in the field, and comparing our proposed method against these establishes a baseline for performance evaluation. The simulation specifics are illustrated below.

Table 2 presents the network parameters taken for simulation. For scalability, the network was designed with 30, 40, 50, 60, 70, 100, 200, and 300 nodes. Of the 300 nodes, 10% were designated the beacon nodes, and different communication radii were taken to represent the transmission power of the node.

### 4.1. Performance Metrics of Algorithm

The metrics used for the evaluation of the proposed algorithm, CSWCL, against other localization algorithms are outlined below:

**Localization Error (LE) (%):** Localization error specifies the deviation in the location between the calculated position and the actual position of the unknown node in the network. The way to compute the localization error of an unknown node ‘UN’ is given by the Equation (11).

(11) $$LE=\sqrt{{\left(EX_{un}-AX_{un}\right)}^{2}+{\left(EY_{un}-AY_{un}\right)}^{2}}$$

EX_un_, EY_un_ = calculated position of unknown node.

AX_un_, AY_un_ = actual position of unknown node.

**Average Localization Error (ALE) (%):** ALE of the entire network is given by the ratio of the sum of localization error of all the unknown nodes in the network to the total number of unknown nodes in the network. ALE is calculated by Equation (12).

(12) $$ALE=\sum_{i=1}^{k}\frac{\sqrt{{\left(EX_{un}-AX_{un}\right)}^{2}+{\left(EY_{un}-AY_{un}\right)}^{2}}}{K*r}$$

### 4.2. Result Analysis

#### 4.2.1. Average Localization Error vs. Communication Radii

**Figure 4:**
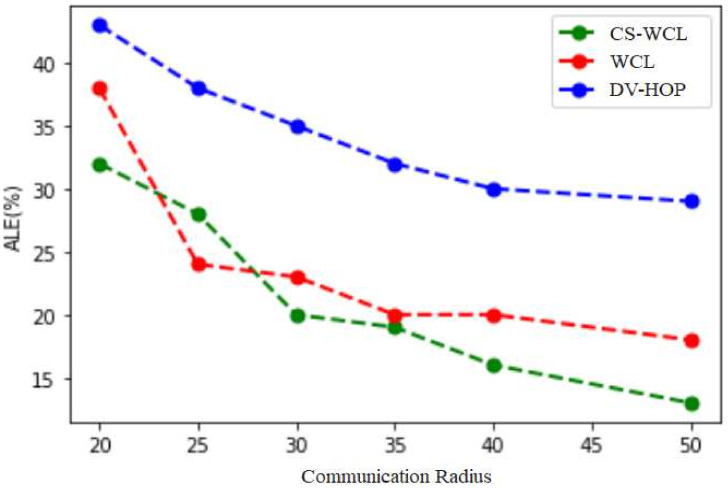
ALE (%) for various ranges of communication radii.

The ALE caused by changing the node′s communication radius is shown in Figure 4. For the communication ranges of 20 m and 45 m, the ALE for CS-WCL noted was 32% and 15%, respectively. The reason for the reduced ALE in CS-WCL is that the relevant anchor nodes were selected by crow search within the communication radius. Regarding DV-Hop and weighted centroid for the same communication ranges of 20 m and 45 m, the ALE computed was greater when compared to that for CS-WCL, although the LE decreased with the nodes’ communication radii, as shown in Table 3. For the communication radii of 20 m and 25 m, the ALE drastically reduced in CS-WCL because the nodes were placed randomly in the region. The main reason for the increased ALE in DV-Hop and WCL when compared with the proposed CS-WCL is that in DV-Hop, only the DV is considered during the localization process. In WCL, although the weighted centroid of all beacon nodes is considered, it is not optimal because the beacon nodes are selected without any specific metric. Overall, when the ALE was compared for all the algorithms with respect to the increase in communication radii, the proposed CS-WCL performed better. Because more neighbor nodes can participate in the localization process with a larger communication range, position estimates will be more accurate. The same is shown in Figure 2.

#### 4.2.2. Average Localization Error vs. Scalability

**Figure 5:**
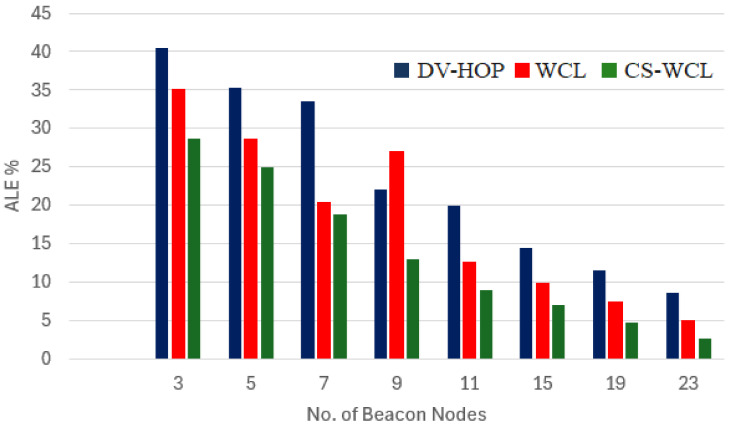
ALE (%) by varying the number of beacon nodes in the network.

The performance of the proposed localization algorithm (CS-WCL) was analyzed concerning the ALE versus scalability of the beacon selection in the network. When the number of nodes increased, the LE also increased. The ratio of beacon nodes in the network plays a vital role in minimizing the LE for unknown nodes. From Figure 5, it is obvious that when the number of beacon nodes increased, the CS-WCL performed better in terms of the LE. From Table 4, it can be seen that for a network setup with 23 randomly selected beacon nodes, the ALE for the localization of unknown nodes was 8.57% and 5.04% for DV-Hop and WCL, respectively. For CS-WCL, the ALE reduced to 2.59%. The CSA used beacon nodes to guide the optimization process, where the efficiency of the beacon location is critical in accurately localizing unknown nodes. If the beacon nodes are not correctly located, the optimization process may converge to a sub-optimal solution, reducing the algorithm’s accuracy and efficiency. To ensure the algorithm’s efficiency, it is necessary to select and locate the beacon nodes correctly, covering the entire search space and allowing efficient exploration of the optimization landscape.

#### 4.2.3. Total Data Packets

**Figure 6:**
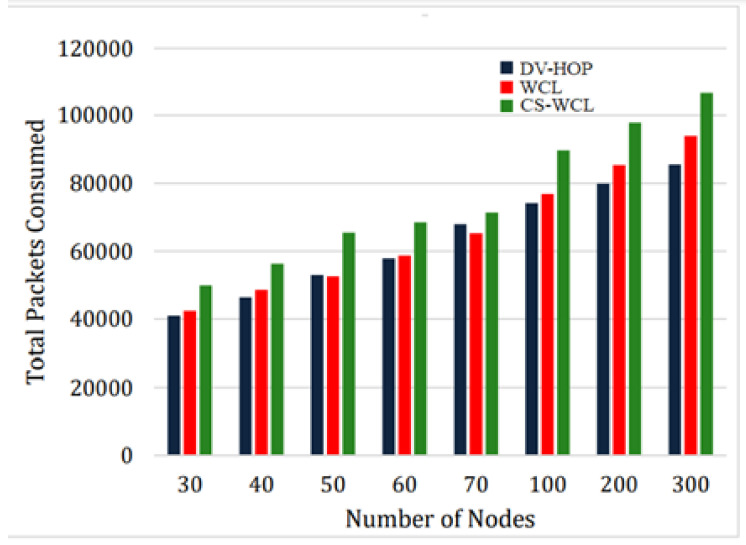
Total number of data packets consumed.

**Figure 7:**
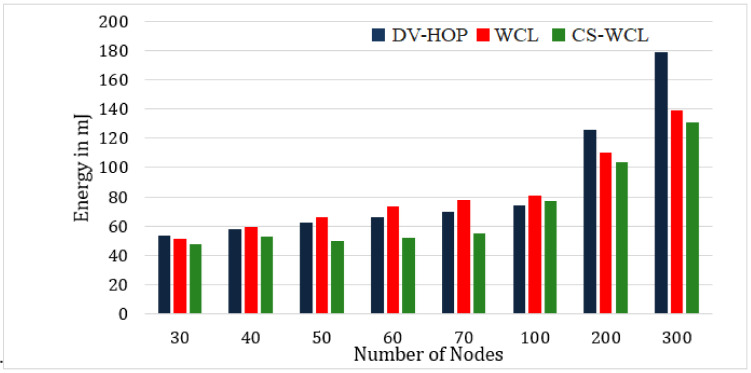
Energy consumption of the nodes.

In the proposed CS-WCL, beacon nodes can communicate with nearby nodes if they are under a specified threshold limit. The DV-Hop localization technique involves the participation of all beacon nodes in the network, which reduces the need for pre-communication control packets. However, with CS-WCL, only the beacon nodes that fall inside the communication radii are considered for localizing unknown nodes. This is evident in the work’s performance, as depicted in Figure 6. Out of the 300 nodes in the network, around 7% of them were chosen as beacon nodes. Around 100,000 packets were used in the process of identifying an unknown node. In some instances, there was a slight rise in packet consumption, but it was insignificant when compared to WCL. This was due to the need for extra communication packets during the beacon selection process. CS-WCL enables the WSN to identify the most effective subset of beacon nodes that can optimize network performance while minimizing energy usage. CS-WCL achieves energy efficiency by optimizing the selection of beacon nodes, which results in reduced energy usage even with a higher number of packets being used. CS-WCL exhibited the lowest energy use, as depicted in Figure 7, despite having the largest total number of data packets consumed, as depicted in Figure 6.

#### 4.2.4. Energy Consumption of the Nodes

The average energy consumed by the nodes in various scenarios was computed and compared, and the efficiency of the CS-WCL approach was demonstrated in terms of energy consumption in every scenario, making it suitable for deployment. The suitability of the CS-WCL approach for deployment was indicated by its efficient performance in every scenario, and the importance of efficient energy consumption for the long-term success of node localization in WSNs was also emphasized. The use of approaches like crow search optimization for beacon selection optimized the energy consumption. The main idea behind the CS-WCL is minimizing the energy needed for the localization of the unknown sensor nodes in a network. The energy utilized for the localization process was measured in mJ. Since the number of communication packets used for localization using the proposed algorithm was fewer, the energy consumed also decreased. The energy needed for the overhead of the localization process was less when our proposed algorithm was used than when the DV-Hop and WCL algorithms were used. To the overall network, the average energy consumption was 125 mJ, 105 mJ, 75 mJ, 45 mJ, 47.53 mJ, 50.48 mJ, 52.21 mJ, and 55 mJ, respectively, for the 30-, 40-, 50-, 60-, and 70-node network setups as displayed in Figure 7.

In addition to validating the proposed crow search weighted centroid localization against WCL and DV-HOP through MATLAB simulation, we also performed a comparative analysis of the proposed algorithm, namely the crow search weighted centroid algorithm, against other meta-heuristic localization algorithms for WSN. These are tabulated in Table 5.

#### 4.2.5. Discussion

The proposed CS-WCL was simulated using MATLAB and validated against DV-HOP and WCL algorithm towards node localization against different metrics, namely the localization error, beacon nodes, total data packets consumed, and energy consumed. This was carried out for network size of 100 × 100 m^2^ with nodes ranging from 30 nodes to a maximum of 300 nodes.

The proposed CS-WCL improvised over other natural inspired algorithms employed for localization where the best three anchor nodes are selected. In addition, the selected anchor nodes participated in the localization process using the weighted centroid method to estimate the location of the unknown nodes. This algorithm aims to provide a more accurate localization solution for unknown nodes in WSNs by considering the trade-off between searchability and the change of awareness probability.

From simulation results, CS-WCL achieved a reduced localization error against communication radii as compared to DV-HOP and WCL which ranged from 32% to 15% for communication radii from 20 m to 45 m for a 30–300 node count. The reason is due to more neighbor nodes participating in the localization process with a larger communication range, resulting in more accurate position estimates. This is not the case with DV-HOP as the distance vector was only considered, and in WCL, the weighted centroid considered for all beacon nodes was not optimal as beacon nodes were selected without any specific metric.

In terms of average localization error vs. scalability of beacon node selection in a network, CS-WCL outperformed DV-Hop and WCL. From the simulation, as shown in Table 4, it was proved that for a network setup of 23 randomly selected beacon nodes, the ALE for CS-WCL was 2.59% as compared to DV-HOP and WCL, which were 8.57% and 5.04%. This is due to fact that the beacon nodes guide the optimization process where the efficiency of the beacon location is critical in accurately localizing unknown nodes, resulting in efficient algorithm performance.

In terms of total data packets consumed for CS-WCL against DV-HOP and WCL, it was found that for a network size of 300 nodes, 100,000 packets were used in identifying the unknown nodes, which was slightly more than DV-HOP and WCL in some instances. For 300 nodes, as per the analysis, only 7% were chosen as beacon nodes. This was due to the need for extra communication packets during the beacon selection process. CS-WCL enables the WSN to identify the most effective subset of beacon nodes that can optimize network performance while minimizing energy usage. CS-WCL exhibited the lowest energy use, as depicted in Figure 7, despite having the largest total amount of data packets consumed, as depicted in Figure 6.

Finally, the energy consumption of nodes towards localization was analyzed for CS-WCL against DV-HOP and WCL. From the analysis, it was found that the average energy consumption was 125 mJ, 105 mJ, 75 mJ, 45 mJ, 47.53 mJ, 50.48 mJ, 52.21 mJ, and 55 mJ, respectively, for the 30-, 40-, 50-, 60-, and 70-node network setups as displayed in Figure 7. It was found from the analysis that the energy needed for the overhead of the localization process was less as compared to DV-HOP and WCL. Since the number of communication packets used for localization using the proposed algorithm was less, the energy consumed also decreased.

In addition to the simulation analysis of CS-WCL against DV-HOP and WCL, the proposed CS-WCL has been benchmarked against other localization methods cited in the literature as shown in Table 5. From the benchmark analysis shown in Table 5, CS-WCL achieved a minimal localization error of 15%, which was the least for a network size of 100 × 100 m^2^, compared to other meta-heuristic localizations for the same network size.

In addition, for 300 nodes with a large network size, our proposed algorithm, i.e., CS-WCL, achieved an LE of 15%; in contrast, using other algorithms, the LE achieved was higher for smaller network sizes with lower numbers of nodes. Lastly, our algorithm achieved a low number of beacons (2.59%) for a network size of 100 × 100 m^2^ and 300 nodes; for other meta-heuristics with the same network size and fewer nodes, the number of beacons used was more. A comparison of meta-heuristic localization approaches is shown in Table 5. This clearly shows the improvement our CS-WCL achieved over other localization approaches, which has been benchmarked.

## 5. Conclusions and Future Work

Sensor node localization in WSNs is a challenging task, and localization with optimal beacons is an efficient approach that enhances localization accuracy. There has been quite a significant amount of work performed in terms of localization using biological inspired algorithm, which does pose challenges pertaining to higher localization errors and a greater number of beacon nodes for small- to large-scale networks.

Accordingly, the crow search weighted centroid localization algorithm was employed, which consisted of two phases. The first phase involved optimizing anchor node selection using the crow search algorithm, and the second phase was identifying the unknown nodes’ locations using weighted centroid localization. The proposed CS-WCL was evaluated against the DV-HOP and WCL in terms of localization error, total data packets consumed, and energy consumption. From the results obtained, CS-WCL achieved reduced localization error against WCL and DV-Hop. In addition to localization error, the CS-WCL achieved a lower number of beacon nodes and reduced energy consumption for varying nodes from 30 to 300 with a network size of 100 × 100 m^2^. This was compared against other methods, namely DV-Hop and WCL. Lastly, the total number of data packets consumed in CS-WCL was slightly more for different network nodes against DV-HOP and WCL, which was marginally negligible. This was due to the need for extra communication packets during the beacon selection process. CS-WCL enables the WSN to identify the most effective subset of beacon nodes that can optimize network performance while minimizing energy usage.

Overall, the CS-WCL outperformed WCL and DV-Hop as shown in graphs and tables.

In addition, the CS-WCL was compared against other localization approaches mentioned in the literature in terms of localization error and the number of beacon nodes for the number of network nodes with different network sizes. The analysis clearly highlights that CS-WCL has far better improvement than other approaches mentioned in the literature for a greater number of nodes with large network sizes, resulting in precise localization.

In future, a WSN testbed would be created to pick the anchor node while simultaneously localizing mobile and stationary nodes alike. Also, the CS-WCL localization would be integrated with data aggregation and fault detection.

In addition, the CS-WCL would be incorporated in 3D wireless sensor networks for more precise localization. For the traditional two-dimensional localization system to be able to function in a three-dimensional environment, it would be necessary to update it. It is feasible that the utilization of sensor nodes that are dispersed across all three dimensions will lead to the development of more precise methods of localization. It is feasible that the usefulness and application of CS-WCL will be increased because of this expansion over a greater variety of scenarios that occur in the real world. This expansion will cover a better range of situations.

## Figures and Tables

**Table 1:** Summary of previous works.

Reference	Localization Approach	Improvements	Research Findings	Challenges
Huang et al. [20]	MA*-3DDV-Hop	3D Accuracy, NSGA-II Optimization	Overcoming Convergence Issues, Effective Memory Usage	Increased computing burden for optimization algorithms
Sharma et al. [21]	Line Search and Genetic Algorithm	Optimal Hop Size, Distance Error Optimization	Enhanced Accuracy with Selected Anchor Nodes, Increased Computing Burden	Increased computing burden for optimization algorithms
Messous et al. [22]	Enhanced Recursive DV-Hop	Polynomial Coefficients, Error Correction	Improved Connection between Hops and Distances, Challenges in Unpredictable Behavior	Unpredictable behavior with random anchor node selection, increased computation complexity
Jondhale et al. [23]	Adaptive Weighted Centroid Localization (A-WCL)	LQI Difference, Quadratic Weights	Precision Increase without Complex WCL Calculation, Drastic LQI Cutoff Changes	Drastic changes in cutoff for minimum LQI with changing communication ranges
Saad et al. [24]	Dynamic Weighting Parameters	RSSI Consideration, Global Error Correction	Sub-Region Adaptation for Accuracy Increase, Challenges in Obtaining Global Factors	Difficulty in obtaining global factors in scenarios with various sub-regions
Kaur et al. [25]	Modified Weighted Centroid Localization (MWCL)	Weight Structure Modification	Adjusting Degree for Best Localization Accuracy, Challenges in Influence Determination	Uncertain influence determination by adjusting weights
Han et al. [26]	Movable Anchor with GPS	Geometric Coordinates Broadcasting	Various Localization Techniques Developed Based on Mobile Anchor Node	GPS Hardware required
Singh et al. [27]	Range-Free Localization	Geometric Theory on Virtual Circle	Chord-Based Estimation, Iterative Procedure, Improved Localization Precision	Limited Accuracy in Dense Networks.
Singh et al. [28]	Geometric Constraint-Based Localization	Intersection of Anchor Coordinates, Average Junction Points	Limited Area Minimization, Enhanced Localization Precision	Sensitivity to measurement errors, limited robustness.
Luo et al. [29]	Maximum-Likelihood, Min-Max, Trilateration	Utilizes Anchor Position and Distance, K-Means Clustering	Decreased Computing Time, Challenges in NP-Hard Problems	NP-hard localization problem
Draz et al. [30]	Trust-Based Beacon Node Localization	Trust-Based and Nature-Inspired Meta-Heuristics (PSO, ACO)	Improved Security, Reliability of Localization, Nature-Inspired Strategies Effective	Impact of false or malicious nodes
Nain et al. [31]	Error Minimization Protocol	RSS and TOA for Range Estimation, LOS/NLOS Signal Consideration	Reduced Localization Errors for NLOS Signals, Addressing Misclassification	Addressing misclassification problem
Cheng et al. [32]	MAOADV Distance Vector Hop	Chaotic Mapping, PSO, Archimedes Optimization	Global Convergence, Speed Enhancement, Challenges in Initial Population Diversity	Sensitivity to Parameters.
Chen et al. [33]	GADV-Hop (Genetic Technique Distance Vector Hop)	Genetic Algorithm, Viable Region Limitation	Faster Convergence, Precision Improvement, Improved Quality of Starting Population	Insufficient Information for Optimal Convergence

**Table 2:** Simulation parameters.

Simulation Parameters	Value
Network region	100 m × 100 m
Overall node count	30, 40, 50, 60, 70, 100, 200, 300
Total number of beacon nodes	3, 5, 7, 9, 11, 15, 19, 23
Communication radii of the node	20, 25, 30, 35, 40 and 45 m
Data packet size	512 bits
Topology	Random

**Table 3:** Average localization error.

	ALE (%)		
Communication Radius (in Meters)	DV-Hop	WCL	CS-WCL
20	47.5	38	32
25	38	24.6	28.5
30	35.26	24	20
35	32	22.4	19.6
40	31.5	21	17.85
45	31	19	15

**Table 4:** Average localization Error on the number of beacon nodes.

	ALE (%)		
No. of Beacon Nodes	DV-Hop	WCL	CS-WCL
3	40.5	35	28.75
5	35.26	28.58	25
7	33.49	20.36	18.78
9	22	26.9	13
11	20	12.57	9
15	14.47	9.8	6.97
19	11.52	7.42	4.78
23	8.57	5.04	2.59

**Table 5:** Comparison of meta-heuristic localization approaches.

Title	Methods	Network Size	No. of Nodes	Beacon Nodes	Summary	ALE
“Node Localization Algorithm Based on Modified Archimedes Optimization Algorithm in Wireless Sensor Networks” [35]	The Modified Archimedes Optimization Algorithm (MAOA)	100 × 100 m^2^	80–200	20%	In wireless sensor networks, optimization reduced distance measurement errors and made use of restrictions to identify the best node positions.	38% to 25%
“PSO-Based Target Localization and Tracking in Wireless Sensor Networks” [36]	RSM- PSO (Region segmentation method)	100 × 100 m^2^	100	-	To increase positioning and tracking speed while maintaining target localization and tracking accuracy, the RSM approach decreased PSO algorithm particles.	21%
“Node Localization in Wireless Sensor Networks Using Butterfly Optimization Algorithm” [37]	Butterfly Optimization Algorithm, Firefly Algorithm, PSO	100 × 100 m^2^	25–150	10–35	A comparison of all three showed that BOA, both in terms of accuracy and computation time, performed significantly better than other algorithms utilized in this study.	20% to 76%
“Optimized Approach for Localization of Sensor Nodes in 2D Wireless Sensor Networks Using Modified Learning Enthusiasm-Based Teaching–Learning-Based Optimization Algorithm” [9]	Modified learning enthusiasm-based teaching–learning-based optimization (mLebTLBO) algorithm	15 × 15 m^2^	20	3	Applied to a 2D localization issue that had movable target nodes and an exclusive anchor node.	21%
Crow Search Weighted Centroid Localization Algorithm (Proposed)	Crow search optimization	100 × 100 m^2^	300	3–23	CSO was used to choose the beacon, and the weighted centroid was then utilized to identify the unknown nodes.	15%

## Data Availability

Data are contained within the article.
